# Neither vitalist nor mechanist, neither dualist nor idealist: Plessner's third way: Essay review of Helmuth Plessner, *Levels of Organic Life and the Human: an Introduction to Philosophical Anthropology*, New York: Fordham University Press, 2019

**DOI:** 10.1007/s40656-021-00421-7

**Published:** 2021-05-21

**Authors:** Francesca Michelini

**Affiliations:** Institut Für Philosophie, Universität Kassel, Henschelstr. 2, Room 2143, 34127 Kassel, Germany

The English translation of Helmuth Plessner’s (1892–1985) volume *Levels of the Organic Life and the Human. An Introduction to Philosophical Anthropology* (1928; henceforth: LO) finally fills in a long-lamented gap. An English translation already existed of other important essays by Plessner, such as *Laughing and Crying: A Study of the Limits of Human Behaviour* (1941; Eng. 1970); *The Limits of Community. A Critique of Social Radicalism* (1924; Eng. 1999); and more recently (in 2018) of *Political Anthropology* (originally published in 1931 as *Macht und menschliche Natur*), but not of this text, which is undoubtedly his masterpiece, besides being a great classic of 1920s German Philosophical Anthropology. While its programmatic intentions and goals are perfectly in line with that constellation of thought—which had Max Scheler and Arnold Gehlen as its leading figures—Plessne’s work is highly original. For a set of reasons that I will try to elucidate, it also remains highly relevant today.

At the time of its initial publication in 1928, however, the book somehow went unnoticed. Reasons for this can be found, as Plessner himself explains, in that Scheler—who precisely in 1928, shortly before his death, published his famous *The Human Place in the Cosmos*—accused Plessner of having plagiarized his ideas. Furthermore, around that time a great hype had developed around existentialism, as in 1927 Heidegger had published his magnum opus *Being and Time*. Generally speaking, this seems to have led to the relegation of Plessner’s philosophy of life to oblivion (LO, p. xix–xx).

Nevertheless, being deeply convinced of the importance and relevance of his ideas, Plessner published his book again many years later, in 1965, without making any substantial changes to the text, with the exception of a new foreword and an appendix, in which he mentions some recent scientific discoveries which he takes to corroborate his theory (LO, pp. 323–336). The book was then republished a third time in 1975 with no further changes. It is this last edition, which has been translated with philological accuracy by Millay Hyatt (with editorial assistance by Phillip Honenberger) for Fordham University Press with the support of the Plessner Gesellschaft and of the Goethe Institut.

Plessner’s work belongs to the complex, socially and politically troubled context of 1920s Germany. This was nevertheless a particularly lively time for culture, philosophy, and science. Great transformations in European culture already underway since the nineteenth century provided the ground for a new approach in the inquiries on living beings in general and human beings in particular. The *Lebensphilosophie*, under whose umbrella one finds philosophers as different as Henri Bergson, Wihlem Dilthey, Ludwig Klages, and George Misch, is just one of the many strains of thought that had an impact on Plessner’s philosophical views. In addition, ample echo can be found in LO of Gestalt psychology, which rejects a merely mechanistic description of life phenomena, and which, at variance with the prevailing principles of associationism, relies on the key notions of ‘totality’ and of corresponding ‘organised unity’ of knowledge functions.

From within the natural sciences, important signs of renewal came to the fore, enabled among other things by the crisis of some forms of reductionism and, more generally, of the positivist approach, which had prevailed at the end of the previous century. Vitalist positions, embraced in biology by Hans Driesch—Plessner’s zoology teacher—and Jakob von Uexküll, despite their limits, shed light on the distinctive features and autonomy of organisms, and argued against the possibility of fully explaining them in mechanistic terms. Plessner’s text, in this regard, was committed to present an alternative solution to the debate of his time by opposing mechanists as well as vitalists (on this point, see LO, pp. 84–92). The first important tests on the intelligence of primates, carried out by Wolfgang Köhler, had made clear that the category of primates as such was increasingly less suited to support the idea of a constitutive difference between animals and human beings. It is in this varied and complex context that German philosophical anthropology developed with the aim of investigating the distinctive features of human nature.

Despite this background, one should not think of Plessner’s text as an introduction to philosophical anthropology that focuses exclusively on human beings. On the contrary, in line with the assumptions of German philosophical anthropology, human beings are just one part of the great web of life. Accordingly, in order to grasp their peculiarities, one should first and foremost understand the peculiarity and autonomy of life itself. In this way, Plessner undertakes an inquiry on living beings in general because he considers this to be as a necessary first step in any investigation of anthropology: “We must begin by clarifying what can be described as being alive before further steps are taken to develop a theory of the experience of life in its highest human layer” (LO, p. 32). This project is reflected by the structure of the book itself, since reference to human beings is only made in the last of its seven chapters.

It is clear that the inquiry on what life is must take into account the results of science and have these as its starting point. Plessner is well positioned to support this view as, in parallel to philosophy, he also studied zoology. His thinking developed in close exchange with the main scientific theories of his time.^1^ However, one should not make the mistake of confusing the methods and goals of science with those of philosophy. The approach, method, and aim of a ‘philosophical biology’ are, for Plessner, irreducible to the parameters of the natural sciences.

Life cannot be grasped merely in terms of measurements, verifications, and analyses. Nor should the philosophical inquiry stop at the mere elucidation of the findings of science. Philosophy must constitute the background or presupposition of the natural sciences. The core of Plessner’s investigations can be defined as ‘biohermeneutics’. This amounts to claiming that the conceptual articulation of the kind of ‘evidence’ concerning biological phenomena cannot be corroborated nor falsified by empirical knowledge, as it is rather assumed by the natural sciences as an interpretative framework. This ‘evidence’ is then the object of intuition or ‘understanding’, but not of ‘explanation’—according to Dilthey’s famous distinction—or demonstration in scientific terms. As Plessner writes: “All content that can only be acquired by intuition is fated to enter into experience without becoming determinable as experience progresses” (LO, p. 111).

Plessner’s answer to the great question, ‘What is life?’ (later famously addressed by Erwin Schrödinger), is different from answers that assume that the definition of life can be fulfilled by a list of empirical features. According to Plessner, it is necessary to identify the essential and irreducible characteristics of the living; ‘irreducible’ meaning that they cannot be referred back to other primary qualities (LO, p. 107). While borrowing the expression used by the physiologist and physicist Hermann Helmholtz (1821–1894), Plessner calls these characteristics “organic modals” (LO, p. 100). These are, for instance, development, aging and death, systemic character, self-regulation, organization, temporality, and so on. What is at stake here are also empirical features. However, they cannot be evinced by means of a purely empirical investigation, as it is necessary to assume an ‘a priori foundation’. What this means is that it is necessary to identify a distinctive criterion of vitality that can work as a starting point for the deduction of all the irreducible qualities pertaining to the organic. Such a criterion is not something vitalistic or metaphysical but is rather linked to our perception, and rests, according to Plessner, on the relation entertained by organic bodies with their boundary (*Grenze*, in German).

According to Plessner, a spatial object that appears in its own ‘dual aspect’ (*Doppelaspekt*) of inside and outside must at the same time display a boundary between the two, a boundary that belongs equally to the object: “Physical objects of intuition for which a fundamentally divergent relationship between outer and inner objectively figures as part of their being are called *living*” (LO, p. 84). In the case of merely physical bodies, or inanimate things, the boundary is simply identical to the border or outline of the physical body. It belongs neither to the simple body, nor to the surroundings (in Plessner’s words: the *medium*), or perhaps, in a certain sense, it belongs to both of them. The border is actually a simple, virtual ‘in-between’ (*das Zwischen*) between the body and the medium which comes from the reciprocal self-limiting of body with its surroundings. On the other hand, in the case of living things, the boundary does not just mark where they stop and the adjoining ‘medium’ begins; rather, the boundary and the overstepping of that boundary both belong to the body itself (LO, p. 95f.). Living things appear as things that embody boundaries and cross those boundaries.

Plessner’s technical term for this basic category of life is ‘positionality’ (LO, p. 118f.). A living being does not simply fill a space, but rather takes its place. Positionality is not a purely spatial concept; it is rather, as Marjorie Grene has explained, “aquestion of the whole way in which an organism *takes its place* in an environment, arises in it, is dependent on it, yet opposes itself to it” (Grene, 1966, p. 254). Although there is no doubt that a living being occupies space, “its center is nevertheless not a spatial center, it is a core which transcends spatiality and at the same time controls the spatiality of the body whose core it is” (Grene, 1966, p. 263).

Positionality is also not a static concept, unlike what the word might suggest, but rather a radically dynamic one. Its basis is in fact a form of dialectics, or better a “radical conflict” for the living being “between the compulsion to close itself off as a physical body and the compulsion to open up as an organism” (LO, p. 202). This ‘conflict’ can be resolved, Plessner says, in two different ways that concern the organization of living bodies. Firstly, in an open form of organization, in which the organism “is immediately incorporated into its surroundings and constitutes a non-self-sufficient segment of the life circle corresponding to it” (LO, p. 203). Secondly, it can be resolved in a closed form of organization, where the integration into the environment is mediated (LO, p. 209), and where the body ultimately functions as a mediating layer between the living being and its environment. Plessner heuristically relates the distinction between open and closed forms to the difference between plants and animals, although he knows very well that at the empirical level such a strong difference does not exist and there are transitional forms between plants and animals (LO, p. 203). The separation between plants and animals is rather a typological distinction. It is a distinction between two opposite idealized typologies and should not be interpreted as a biological taxonomy. Similarly, this reference to ‘levels’ should not be mistaken for a revival of the ancient model of the *Scala naturae.* Plessner’s notion of levels conveys only the idea that the relation of living beings to their environment is mediated in different ways by their different forms of organization. What is at stake are not progressive degrees of excellence or perfection, but rather different levels of development in the positionality pertaining to the lived body.

Human beings, in this respect, do not engender any new positional level. As they belong, like animals, to the level of the closed positional form, “it is clear that the human must physically stay an animal, as excentricity does not enable a new form of organization” (LO, p. 272). Nevertheless, a crucial difference should be mentioned. The closed form of animals stands for an individual that is separated from its environment, endowed with the faculty to feel and to act, and, at the highest degree, to manage its own corporeality. At least the animals which have a developed nervous centre have bodies (*Körper*) endowed with lived body (*Leib*) that can be used. In this respect, the animal organism already displays a distance between itself (i.e., its non-spatial centre) and its embodiment. However, it is not aware of said distance. Focused, or better, absorbed in the ‘here/now’, the animal does not really establish a relationship with itself: “it is a system that refers back to itself, a self, but it is not experience itself” (LO, 267). Its positional form is of a ‘centric’ type. It has a vital centre to which all its experiences are referred; it lives based on this centre but has no experience of it as such. Unlike animals, human beings are aware of the distance separating them from their bodies and can establish a relationship with themselves. Most notably they are beyond themselves. To say it better, human beings present a double distance from their own bodies: “We have not only an inner life distinct from—though not separable from—our physical existence; we stand over against both these, holding them apart from one another and yet together” (Grene, 1966, p. 274). This is how Plessner puts it:

Although the living being on this level is also absorbed in the here/now, lives out of the center, it has become conscious of the centrality of its existence. It has itself; it knows of itself; it notices itself—and this makes it an *I.* This I is the vanishing point of its own interiority that lies “behind” it; it is removed from its own center in every possible execution of life and is the observer of the scene of this inner field; it is the subject-pole that can no longer be objectified or put into the object position. (LO, pp. 269–270)

Plessner defines humans in terms of “excentric positionality”. ‘Excentric’ means that, although inseparably linked to their animal nature, human beings are detached from any localization and are projected beyond themselves. Humans as animals are bodies and have lived bodies. They are able to manage their bodies and direct them while autonomously placing themselves ‘in front of’ the environment. Finally, they know about their condition and the possibilities ensuing from their own existence. It is as if they could see themselves from the outside. However, this condition does not imply any kind of harmony, nor does it imply that, in virtue of this ability of placing themselves outside of themselves, they are in any way ‘superior’ to other animals.

Human beings are instead identified by intrinsic disharmony and are always on the lookout for balance, always precarious and never fully achievable, between being linked to the environment yet being detached from it; between, that is, their natural dimension and their social and cultural world. In reference to the German terminology, humans are in a precarious dialectic between *Umweltgebundenheit* (environment-connectedness) and *Weltoffenheit* (world-openness). The three anthropological laws (‘Natural Artificiality’, ‘Mediated Immediacy’, ‘Utopian Standpoint’) described in the final chapter of the book refer to the several vital features of human life and account for the ambivalent and precarious story of the most restless of all forms of existence.

My short summary of this complex and bountiful text might already give an idea of why Plessner’s theory—long neglected and rediscovered in Germany only after 1990—is worth reading today. In certain respects, the current state of the discourse, in which Plessner’s philosophy is enjoying a revival, resembles the situation in the early twentieth century when it was first presented. The unprecedented advances in the life sciences and biotechnology, especially since the completion of the human genome project at the end of the last century, have had a profound influence not only on the way philosophers understand the scientific study of life but also on the way we address longstanding questions about humanity and our place in nature. One should also consider that, after a long-lasting focus on genes and the evolution of characters across populations, a revival of the category of the living organism and its organization has decidedly come to the fore. Several contemporary developmental and evolutionary biologists and many philosophers of biology recognize that *organism* is a category we can no longer do without (see Toepfer & Michelini, 2016). Although it is unquestionable that genetic inheritance is key to understanding life, it is no longer considered enough for a full understanding of the phenomenon of life and its autonomy. Biology must indeed presuppose the organism as a system that self-determines and maintains itself, that is to say, as an organized unit which acts on the environment, reproduces itself, transmits its characteristics, and is capable of mutations.

In this context, there is no doubt that Plessner’s philosophical paradigm can offer, concerning our understanding of both human beings and more generally of living beings, an important intellectual reference for those working on these key issues, “thus avoiding the implausible extremes of materialist, reductionism and cultural idealism as well as the impossible compromise of mind–body dualism” (Bernstein’s introduction, LO p. lxiv).

With regards to the question of the human condition, human beings are described by Plessner, by virtue of their embodiment, as intimately and indissolubly linked to the whole web of life. This view is at variance not only with the existentialism of his time, in particular Heidegger’s,^2^ but also with the viewpoint of other philosophical anthropologists. What Plessner presents is not an essentially defective being whose deficiencies must be offset by means of technical compensation (see Gehlen’s idea of *Mängewesen*), nor does he believe that to fully understand what it means to be human a metaphysical leap is required, ultimately leading—this is his reproach to Scheler—to a form of theomorphism (LO, p. xiii).

What is distinctive about human beings is not language, their technical and artistic abilities, or any of the features that for centuries intellectuals and philosophers have credited to humans to distinguish them from animals (or God) by means of additive (or subtractive) formulations (claiming, for instance, that human beings are animals *plus* reason). To understand what is peculiar about humans, Plessner argues, one should understand their peculiar positional form, and insist on the relationship between their living body and the surrounding environment. Reason, the objectivating faculty, language, as well as phenomena such as crying and laughing, are possible because humans can place themselves outside of themselves, because, that is, humans have an excentric positional form. Humans are fully contingent beings, with no certainty, homeland, or place. They are displaced beings. Plessner says that “[their] existence is literally based on nothing,” (LO, p. 272), and they are not even linked to a precise morphology: they could exist also in other forms that are not familiar to us.^3^ Already in the 1940s, Plessner believed that his theory of the human could serve as antidote to all theories based on race, as well as to blood and soil (*Blut-und-Boden*) ideologies, which unfortunately, under new forms, are hardly disappearing even today.

A second reason for interest in Plessner’s book today lies, of course, in its more general theory of life, which takes up the main part of LO. According to Plessner, life cannot be understood on an exclusively physical or psychological basis. Life is neither a mysterious matter-pervading force nor something added to bodily reality. It is not a hidden, secret, or deeply concealed quality. Although he does not accept that life can be understood only through mechanistic explanations, this does not mean that its essence is to be found in mysterious vitalistic presuppositions. Plessner does not actually deny the explanatory validity of mechanism. He rather breaks free from the dogmatic dichotomy according to which, in the life sciences, one must either be vitalist or mechanist, and paves the way, instead, to an alternative approach, a ‘third way’.

This position is grounded, as we have seen, in his idea of ‘double aspectivity’ and the realization of the boundary. With this Plessner provides a viable starting point for the elaboration of a theory of the organism that not only eludes any Cartesian dualism, but also overcomes the limits of any simplified organic monism. Against an undifferentiated monism, Plessner maintains the practical advantages of the distinction between physical and mental without entirely questioning its ability to grasp essential features of reality. And, against all form of dualism, between spirit/mind and life, humans and nature, he shows to what extent interiority and subjectivity are part of the phenomenon of life and they must not simply be equated to the sphere of human consciousness or self-consciousness: “A self is not yet a subject of consciousness”, he writes (LO, p. 148).

Some authors (Mugerauer, 2014; Fischer, 2008; Moss, 2020) have pointed out the affinities between Plessner’s main assumptions (e.g. the realization of the boundary) and the theory of autopoiesis, going as far as to say that Plessner seems to have anticipated “central concepts of autopoiesis” (Moss, 2020). Plessner’s description of living organisms as at the same time “both enclosing/shielding and opening/mediating in relation to the surroundings” (LO, p. 332) sounds indeed similar to the main defining feature of autopoietic systems as being at the same time organizationally closed and thermodynamically open. Plessner died in 1985 and he did not have the opportunity to get directly acquainted with the autopoietic theory, which he might have considered almost an “empirical confirmation” of his ideas.^4^

There is, however, one non-secondary conceptual difference between the two approaches. According to first-generation autopoiesis theorists (Maturana & Varela, 1980), the interaction with the environment plays a marginal role in the definition of the autonomy of the system—the interactions with the environment are seen as ‘structural couplings’. In contrast, according to Plessner, the core meaning of the autonomy of the living is precisely its ‘positioning’ in relation to the environment, without which the organism is only “the half of its life” (LO, p. 180). In this regard, his philosophy of the living could today be compared, probably more effectively, to the theories of life and mind which have worked on overcoming the difficulties entailed by the first formulations of autopoiesis, notably with the proposals of embodied cognition, enactivism, and, in general terms, the theories of autonomy in biology (see, in particular, Moreno & Mossio, 2015). The ‘contamination’ of enactivist positions with Plessner’s theory might even produce the favourable results of widening its range and freeing it, for instance, from its prevailing focus on the individual organism—this is Plessner’s greatest limit, according to, for instance, Lenny Moss (2020). Future inquiries that wish to overcome this limitation should no doubt take into account such factors as the “relation between competitive and altruistic forms of behaviour in evolution”, the “complexity of microbial life”, and “the ubiquity of symbiosis” (Bernstein’s introduction, LO p. lxv).

To conclude this review, a few remarks on the new edition of this important book are due. Generally speaking, the restitution of the text in English appears both fluid and faithful to the original. Terminological choices are, on the whole, compelling. The volume also presents a glossary of Plessner’s most used terms, both English to German and vice versa, which helps readers navigate their way around the complex technical terminology (LO, p. 337–343). In some instances, as it is almost unavoidable in translations, the choice in favour of consistency with the original is to the detriment of contextual meaning. Just to give one example, the lexical choice to always consistently translate *Umwelt* as ‘environment’ and *Umgebung* as ‘surroundings’, although fully understandable, is not altogether compelling, since it is often the case that in Plessner’s writing *Umwelt* and *Umgebung* overlap to indicate the same thing, that is, the environment in general. A clear distinction can instead be found in the theoretical biology of Jakob von Uexküll, who strongly influenced Plessner. As is well known, Uexküll distinguished the *Umwelt* as what stands for the subjective perspective of the animal on the environment from the *Umgebung* as what happens in the whereabouts of a living being; in other words, as what the human being as observer perceives as the generic environmental surrounding of the living being. Plessner adopts this distinction when he is referring directly to Uexküll’s philosophy. However, unlike Uexküll, he tends to use different words depending on the living being he is dealing with. For instance, concerning plants, Plessner seems to systematically (although not always) use the word *Umgebung* meaning environment. An additional complication ensues when, concerning some animals, the word *Umfeld* is used, translated as ‘surrounding field’ this edition, to indicate the environment in which the existence of the animal organism is placed. This is just one example of the many difficulties entailed by the translation of such a complex work, concerning which one cannot but sympathize with Millay Hyatt and Phillip Honenberger when, in the translator’s preface to the text, they say: “Rendering Plessner’s magnum opus into English has been a daunting and rewarding task” (LO, p. IX).

Besides the translation, the order of the materials could have been better. J. M. Bernstein’s Introduction should have been placed at the beginning, after the Translator’s Preface and the Acknowledgements, and not after Plessner’s two Prefaces (1928/1965), as the current order breaks the continuity between the prefaces and the first chapter, which are all original parts of Plessner’s text. A similar problem afflicts the Appendix, added by Plessner in 1965 and integral to the book. As in the Table of Contents its title is given in italics, one might fail to distinguish Plessner’s actual contributions to the volume from the subsequent editorial additions.

These quibbles notwithstanding these details, the idea of making LO finally accessible in English translation is certainly to be commended. Hopefully, it will give raise to the wide international discussion it deserves, not only within philosophical anthropology but also in philosophy of biology. Plessner’s work opens new perspectives for the future, which “should be taken as exemplary of the kind of undertaking biophilosophy is and must become”, as Bernstein writes in the Introduction. “If human beings are living beings”, he adds, “then the fact that the philosophy of biology is a marginal subdiscipline speaks to a massive distortion in contemporary philosophy” (LO, p. lxv). Reading Plessner’s work can certainly contribute to correct this distortion.

## Footnotes

1 A review of the several definitions of life given by contemporary scientists—such as Wilhelm Roux and Adolf Meyer—can be found in LO, Chapter 3, § 7. On Plessner and the scientific theories of his time—particularly those of Jakob von Uexküll, Wolfgang Köhler, and Hans Driesch—see Köchy and Michelini (2015).

2 From a general point of view, Plessner sees in Heidegger one of the latest configurations of the dualism-based philosophical tradition that fails to investigate nature—and in particular the living organism—in its ‘immense concatenation’ with the human world, hence abandoning this latter to the dominion of biology and science (Plessner, 2003, p. 45). On the relation between Plessner and Heidegger, see Michelini (2019).

3 “Being human is not tied to any particular gestalt and (to recall an imaginative conjecture by the paleontologist Edgar Dacqué) could just as well take on a variety of gestalts that do not correspond with our own” (LO, p. 272).

4 In the sixties, Plessner presents his theory as corroborated by the back then recent findings on membranes’ semipermeability, including a direct reference to I.B.S. Haldane (LO, p. 332). More empirical evidence could be found in the theory of protoplasm and of immune system (on this point, see Grene, 1966, p. 255, and Mugerauer, 2014).
